# Decoding heterogeneous electrocatalysts for acidic oxygen evolution: mechanisms, rational design and AI acceleration

**DOI:** 10.1093/nsr/nwaf474

**Published:** 2025-11-08

**Authors:** Xingen Lin, Wenjia Qu, Zihan Wang, Jianuo Liu, Cheng Qian, Lin Tian, Lingxiao Wang, Yu Zhang, Huang Zhou, Yafei Zhao, Yuen Wu

**Affiliations:** State Key Laboratory of Precision and Intelligent Chemistry, School of Chemistry and Materials Science, University of Science and Technology of China, Hefei 230026, China; Deep Space Exploration Laboratory, University of Science and Technology of China, Hefei 230026, China; Tianjin Key Laboratory of Advanced Carbon and Electrochemical Energy Storage, School of Chemical Engineering and Technology, and Collaborative Innovation Center of Chemical Science and Engineering (Tianjin), Tianjin University, Tianjin 300072, China; State Key Laboratory of Precision and Intelligent Chemistry, School of Chemistry and Materials Science, University of Science and Technology of China, Hefei 230026, China; Deep Space Exploration Laboratory, University of Science and Technology of China, Hefei 230026, China; State Key Laboratory of Precision and Intelligent Chemistry, School of Chemistry and Materials Science, University of Science and Technology of China, Hefei 230026, China; Deep Space Exploration Laboratory, University of Science and Technology of China, Hefei 230026, China; State Key Laboratory of Precision and Intelligent Chemistry, School of Chemistry and Materials Science, University of Science and Technology of China, Hefei 230026, China; Deep Space Exploration Laboratory, University of Science and Technology of China, Hefei 230026, China; State Key Laboratory of Precision and Intelligent Chemistry, School of Chemistry and Materials Science, University of Science and Technology of China, Hefei 230026, China; Deep Space Exploration Laboratory, University of Science and Technology of China, Hefei 230026, China; State Key Laboratory of Precision and Intelligent Chemistry, School of Chemistry and Materials Science, University of Science and Technology of China, Hefei 230026, China; Deep Space Exploration Laboratory, University of Science and Technology of China, Hefei 230026, China; State Key Laboratory of Precision and Intelligent Chemistry, School of Chemistry and Materials Science, University of Science and Technology of China, Hefei 230026, China; Deep Space Exploration Laboratory, University of Science and Technology of China, Hefei 230026, China; State Key Laboratory of Precision and Intelligent Chemistry, School of Chemistry and Materials Science, University of Science and Technology of China, Hefei 230026, China; Deep Space Exploration Laboratory, University of Science and Technology of China, Hefei 230026, China; State Key Laboratory of Precision and Intelligent Chemistry, School of Chemistry and Materials Science, University of Science and Technology of China, Hefei 230026, China; State Key Laboratory of Precision and Intelligent Chemistry, School of Chemistry and Materials Science, University of Science and Technology of China, Hefei 230026, China; Deep Space Exploration Laboratory, University of Science and Technology of China, Hefei 230026, China

**Keywords:** oxygen evolution reaction, design strategies, mechanisms, artificial intelligence, heterogeneous electrocatalysts

## Abstract

Proton exchange membrane water electrolyzers (PEMWEs) are positioned as a transformative technology for renewable energy conversion and storage systems. The acidic oxygen evolution reaction (AOER), serving as the pivotal half-reaction governing overall efficiency, operational stability and system cost in water electrolysis, has become a focal point of contemporary electrochemical research. In this Review, we comprehensively summarize the recent advancements in both noble metal-based (Ir and Ru) and non-noble-metal-based (Mn and Co) heterogeneous electrocatalysts (HEs) for the AOER. The analysis commences with fundamental AOER mechanisms and the key factors that influence them, elucidating critical structure–activity relationships essential for rational catalyst engineering. Subsequently, we systematically evaluate state-of-the-art design strategies and corresponding breakthroughs in catalyst development, followed by a forward-looking perspective on the emergence and application of AI for science in the AOER. This review provides valuable guidance for the design of next-generation HEs for the AOER, ultimately aiming to bridge the gap between laboratory-scale achievements and industrial implementation of PEMWE technologies.

## Teaser

This review highlights recent advances in designing and engineering acidic oxygen evolution catalysts, emphasizing strategies to enhance stability and efficiency for practical hydrogen production in renewable energy systems.

## INTRODUCTION

Hydrogen is regarded as a valuable energy carrier and a promising alternative to traditional fossil fuels due to its sustainable characteristics and environmentally benign nature [1]. Currently, hydrogen is primarily produced through natural gas reforming and electrochemical water splitting. Among these, water electrolysis driven by renewable energy sources has gained increasing attention owing to its sustainability advantages [2]. In the electrochemical water splitting process, the hydrogen evolution reaction (HER) occurs at the cathode through a two-electron transfer mechanism, while the anode undergoes the oxygen evolution reaction (OER) involving a more intricate four-electron transfer process coupled with multiple proton–electron interactions [3–8]. This fundamental difference in reaction mechanisms results in inherently sluggish reaction kinetics and elevated overpotential requirements for the OER compared to the HER [9]. Consequently, the OER has been identified as the rate-determining step (RDS) in water electrolysis systems, constituting a critical bottleneck in overall energy conversion efficiency.

**Figure 1.**
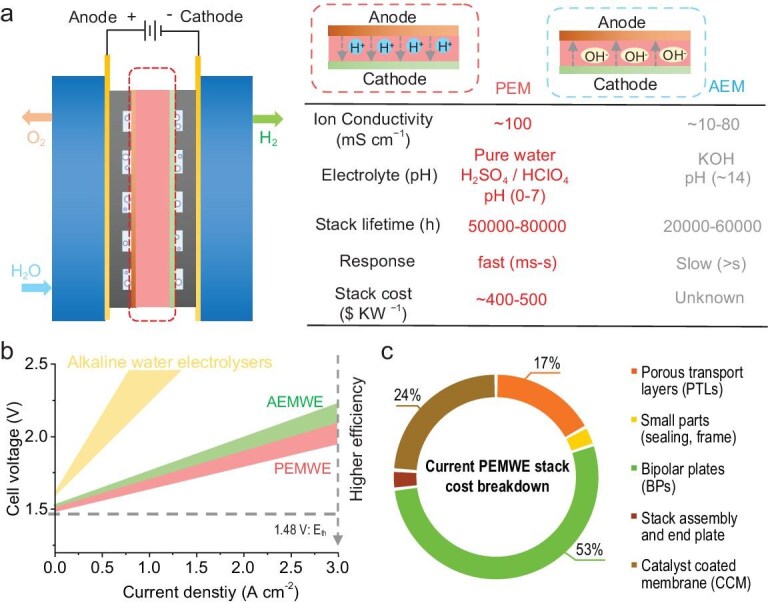
(a) Comparison of key parameters between AEMWEs and PEMWEs. (b) Comparison of typical polarization curve ranges for different types of electrolyzers. (c) Cost breakdown of a PEMWE stack. Adapted with permission from Yuan *et al.* [14]; Copyright 2024, Elsevier.

Recent water electrolysis technologies have primarily focused on proton exchange membrane water electrolyzers (PEMWEs) and anion exchange membrane water electrolyzers (AEMWEs), as they can produce high-purity hydrogen compared to conventional alkaline water electrolyzers [10]. Among these, PEMWEs have a longer development history and exhibit technical maturity in several key parameters (Fig. 1a). For example, commercial advanced proton exchange membranes (PEMs) still offer superior ionic conductivity (∼100 mS cm^−1^) compared with anion exchange membranes (AEMs). The well-established PEM technology also reduces membrane costs and extends the overall stack lifetime relative to AEMWEs. Moreover, the PEMWE electrolyte consists of pure water or acidic aqueous solutions (H_2_SO_4_, HClO_4_), which provides advantages in applications that require strict pH control, compared with AEMWEs that rely on alkaline electrolytes. PEMWEs also demonstrate faster start-up and shutdown response times (typically <10 s) [11], enabling better compatibility with intermittent renewable energy sources such as wind and solar power. According to the International Renewable Energy Agency (IRENA), the state-of-the-art cost for a PEMWE is approximately 400–500$ kW^−^^1^, whereas no cost data are available for AEMWEs due to the lack of commercial cases [12]. In state-of-the-art electrolyzers, the energy efficiency of PEMWEs (1.9 V @ 3 A cm^−^^2^, DOE 2050 target) remains higher than that of AEMWEs and conventional alkaline electrolyzers, making PEMWEs particularly competitive in systems where electricity cost dominates (Fig. 1b).

The advancement of heterogeneous electrocatalysts (HEs) for the acidic oxygen evolution reaction (AOER) has predominantly focused on materials incorporating iridium (Ir), ruthenium (Ru), cobalt (Co) and manganese (Mn) metals. The AOER is indispensable and more preferable for the industrial PEMWEs [13]. Within the PEMWE stack, catalyst cost constitutes a significant fraction due to the use of noble metals such as Ir and Pt (Fig. 1c) [14]. Among these, the anode OER catalyst represents the major portion of both loading and cost. Therefore, the design and optimization of anode OER catalysts are both urgent and essential for improving PEMWE energy efficiency and durability, and reducing overall system costs. However, the AOER process presents significant challenges, particularly with regard to catalyst stability and the high cost associated with noble metal usage. Despite these limitations, ongoing research continues to push the boundaries of material design, composition tuning and mechanistic understanding to overcome these hurdles.

As electrochemical water splitting establishes itself as a cornerstone technology in sustainable energy conversion, the critical role of the AOER as the primary anodic process has intensified scientific investigation. Nevertheless, the rigorous performance demands for activity, durability and cost-effectiveness persist as formidable obstacles in catalyst development. This review systematically examines contemporary breakthroughs in AOER catalyst design, categorizing and analyzing representative strategies across different material systems. Our critical assessment reveals fundamental structure–activity relationships through mechanistic analysis of the AOER process and its governing parameters. The subsequent evaluation of design methodologies for Ir-, Ru-, Mn- and Co-based HEs establishes a framework for optimizing the activity–stability–cost triad in HEs for the AOER. This comprehensive analysis not only synthesizes fundamental mechanistic understanding with cutting-edge material innovations but also provides strategic guidance for advancing next-generation electrocatalyst development. The insights presented herein are anticipated to accelerate the practical implementation of PEMWE technology and related electrochemical energy conversion systems.

## OER MECHANISMS AND REGULATIONS

The OER process involves intricate multi-electron and proton-coupled transfer steps, where divergent reaction pathways yield distinct intermediates and energy landscapes. Therefore, the OER mechanism not only directly governs the intrinsic activity of the catalyst but also critically determines catalyst stability under harsh operating conditions. A deep understanding of these OER reaction pathways is crucial for strategically designing and refining electrocatalyst performance. Recent advancements in spectroscopic characterizations and theoretical simulations have been employed to reveal the dynamic structural transformations in HEs and oxygen-containing intermediates during the OER process [15]. Among the most widely recognized mechanisms are the adsorbate evolution mechanism, lattice oxygen mechanism, and the oxide path mechanism (OPM). In this Review, we systematically discuss these mechanisms and summarize the key factors that have recently been identified to influence the AOER pathways. These insights further contribute to expanding strategies for the design and optimization of HEs for the AOER.

### OER mechanisms

**Figure 2.**
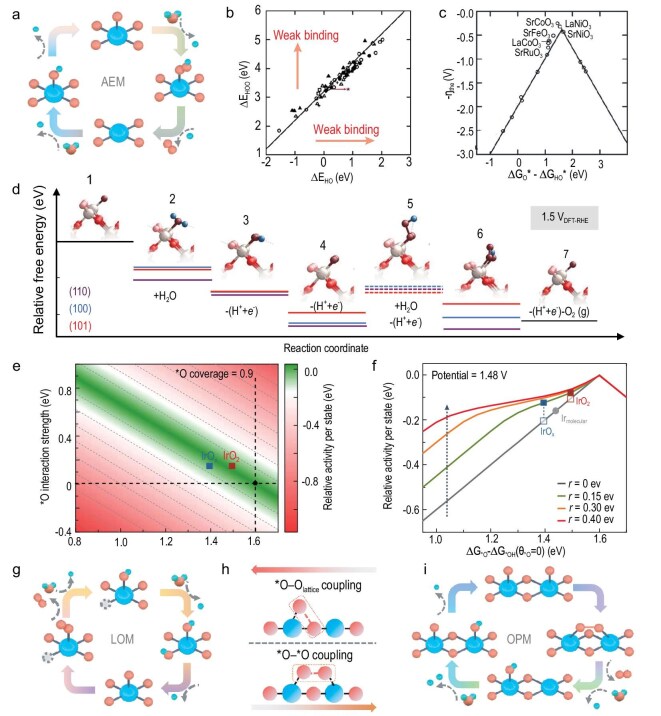
(a) Illustration of the AEM in the AOER. (b) Adsorption energy of *OOH plotted against the adsorption energy of *OH on oxides. (c) Activity trends towards oxygen evolution plotted for perovskites. Adapted with permission from Man *et al.* [18]; Copyright 2011, Wiley-VCH. (d) DFT-calculated free energy diagram for the OER mechanism at 1.5 V_DFT-RHE_, showing the six possible intermediates. Adapted with permission from Rao *et al.* [20]; Copyright 2020, Springer Nature. (e) Contour plot of relative activity as a function of the free energy of *O relative to *OH at an *O coverage of zero. (f) Relative activity per active state at a potential of 1.48 V_RHE_ and the corresponding positions for amorphous IrO*_x_*, rutile IrO_2_ and molecular Ir catalyst. Adapted with permission from Liang *et al.* [21]; Copyright 2024, Springer Nature. (g–i) The illustration of the LOM and the comparison of O–O coupling in the LOM and the OPM.

*Adsorbate evolution mechanism (AEM).* The conventional AEM primarily focuses on water oxidation at single-metal sites, involving water dissociation and concerted proton–electron transfer (CPET) processes (Fig. 2a) [16]. It is typically described by the following steps:


(1) \begin{eqnarray*}
{\mathrm{M\ }} + {\mathrm{\ }}{{\mathrm{H}}}_2{\mathrm{O\ }}\rightarrow {\mathrm{\ M}} {-} {\mathrm{OH\ }} + {\mathrm{\ }}{{\mathrm{H}}}^ + {\mathrm{\ }} + {\mathrm{\ }}{{\mathrm{e}}}^ -,
\end{eqnarray*}

(2) \begin{eqnarray*}
{\mathrm{M}} {-} {\mathrm{OH\ }}\rightarrow {\mathrm{\ M}} {-} {\mathrm{O\ }} + {\mathrm{\ }}{{\mathrm{H}}}^ + {\mathrm{\ }} + {\mathrm{\ }}{{\mathrm{e}}}^ -,
\end{eqnarray*}

(3) \begin{eqnarray*}
{\mathrm{M}} {-} {\mathrm{O\ }} + {\mathrm{\ }}{{\mathrm{H}}}_2{\mathrm{O\ }}
\rightarrow {\mathrm{\ M}} {-} {\mathrm{OOH\ }} + {\mathrm{\ }}{{\mathrm{H}}}^ + {\mathrm{\ }} + {\mathrm{\ }}{{\mathrm{e}}}^ -,
\end{eqnarray*}

(4) \begin{eqnarray*}
{\mathrm{M}} {-} {\mathrm{OOH\ }}\rightarrow {\mathrm{\ M\ }} + {\mathrm{\ }}{{\mathrm{O}}}_2{\mathrm{\ }} + {\mathrm{\ }}{{\mathrm{H}}}^ + {\mathrm{\ }} + {\mathrm{\ }}{{\mathrm{e}}}^ -.
\end{eqnarray*}

Each CPET process requires a high activation energy. The step with the largest free energy barrier is identified as the RDS, which governs the OER activity. Based on theoretical calculations within the AEM, Nørskov and co-workers found that the free energies of steps 2 and 3 (*OH → *O; *O → *OOH) are highly dependent on the type of the metal site and the binding configuration, and vary across different metals and metal oxide surfaces. However, the *OH → *OOH step is typically treated as a constant (3.2 eV) across various metal and metal oxide surfaces (Fig. 2b) [17,18]. As a result, the RDS of the AEM often lies in competition between the deprotonation step (step 2) and the *OOH intermediate formation (step 3), and is expressed as follows [18]:


(5) \begin{eqnarray*}
&&{\eta }_{{\mathrm{OER}}} = \\
&&\left\{ {\displaystyle\frac{{{\mathrm{max}}\left[ {\left( {{\mathrm{\Delta }}{G}_{{\mathrm{*O}}} {-} {\mathrm{\Delta }}{G}_{{\mathrm{*OH}}}} \right),\left( {{\mathrm{\Delta }}{G}_{{\mathrm{*OOH}}} {-} {\mathrm{\Delta }}{G}_{{\mathrm{*O}}}} \right)} \right]}}{{\mathrm{e}}}} \right\}{-} 1.23{\mathrm{\ V}}\\
&&\approx \left\{ {\frac{{{\mathrm{max}}\left[ {\left( {{\mathrm{\Delta }}{G}_{{\mathrm{*O}}} {-} {\mathrm{\Delta }}{G}_{{\mathrm{*OH}}}} \right),{\mathrm{\ }}3.2{\mathrm{\ eV}} - \left( {{\mathrm{\Delta }}{G}_{{\mathrm{*O}}} {-} {\mathrm{\Delta }}{G}_{{\mathrm{*OH}}}} \right)} \right]}}{{\mathrm{e}}}} \right\}\\
&&-\, 1.23{\mathrm{\ V}}.
\end{eqnarray*}

Furthermore, the free energy difference of the deprotonation step (ΔG_*O_−ΔG_*OH_) is used as a descriptor to predict the OER overpotential (Fig. 2c), displaying a volcano-type trend. The minimum overpotential is estimated to be 0.37 V [19]. In the AEM, the scaling relationships constrain the independent optimization of the energy barrier for each single step. Theoretically, excellent OER activity requires a balanced *O adsorption energy that prevents either the *OH → *O or *O → *OOH step from becoming the high-barrier rate-limiting step. Recent studies refined the conventional AEM by considering oxygen intermediates on neighboring sites. Rao and co-workers proposed that when the traditional AEM occurs at coordinatively unsaturated sites (Ru_cus_) on the surface of rutile RuO_2_, the adsorption of OH on adjacent sites can stabilize the formation of *OOH on the Ru_cus_, thereby shifting the RDS from *OOH formation to the final deprotonation of *OH (Fig. 2d) [15,20]. Liang and co-workers also proposed that the coverage of *O intermediates needs to be considered. On the surface of IrO_2_, *O intermediates exhibit repulsive adsorbate–adsorbate interactions. Increasing their coverage weakens their binding, thereby promoting O–O bond formation (Fig. 2e and f) [21].

*Lattice oxygen mechanism (LOM).* The LOM involves an O–O coupling process that includes the participation of lattice oxygen (Fig. 2g), and has been detected through experiments using ^18^O-isotope labeling combined with differential electrochemical mass spectrometry [22]. It can be described by the following steps:


(6) \begin{eqnarray*}
{{\mathrm{O}}}_{\mathrm{L}} {-} {\mathrm{M\ }} + {\mathrm{\ }}{{\mathrm{H}}}_2{\mathrm{O\ }}\rightarrow {\mathrm{\ }}{{\mathrm{O}}}_{\mathrm{L}} {-} {\mathrm{M}} {-} {\mathrm{OH\ }} + {\mathrm{\ }}{{\mathrm{H}}}^ + {\mathrm{\ }} + {\mathrm{\ }}{{\mathrm{e}}}^ -,
\end{eqnarray*}

(7) \begin{eqnarray*}
{{\mathrm{O}}}_{\mathrm{L}} {-} {\mathrm{M}} {-} {\mathrm{OH\ }}\rightarrow{\mathrm{\ }}{{\mathrm{O}}}_{\mathrm{L}} {-} {\mathrm{M}} {-} {\mathrm{O\ }} + {\mathrm{\ }}{{\mathrm{H}}}^ + {\mathrm{\ }} + {\mathrm{\ }}{{\mathrm{e}}}^ -,
\end{eqnarray*}

(8) \begin{eqnarray*}
{{\mathrm{O}}}_{\mathrm{L}} {-} {\mathrm{M}} {-} {\mathrm{O\ }}\rightarrow{\mathrm{\ }}{{\mathrm{V}}}_{\mathrm{O}} {-} {{\mathrm{M}}}^*{\mathrm{\ }} + {\mathrm{\ }}{{\mathrm{O}}}_2,
\end{eqnarray*}

(9) \begin{eqnarray*}
{\mathrm{V_O}} {-} {{\mathrm{M}}}^*{\mathrm{\ }} + {\mathrm{\ }}{{\mathrm{H}}}_2{\mathrm{O\ }}\rightarrow{\mathrm{\ M}} {-} {\mathrm{OH\ }} + {\mathrm{\ }}{{\mathrm{H}}}^ + {\mathrm{\ }} + {\mathrm{\ }}{{\mathrm{e}}}^ -,
\end{eqnarray*}

(10) \begin{eqnarray*}
{\mathrm{M}} {-} {\mathrm{OH\ }}\rightarrow {\mathrm{\ }}{{\mathrm{O}}}_{\mathrm{L}} {-} {\mathrm{M\ }} + {\mathrm{\ }}{{\mathrm{H}}}^ + {\mathrm{\ }} + {\mathrm{\ }}{{\mathrm{e}}}^ -,
\end{eqnarray*}

where V_O_ denotes oxygen vacancies and O_L_ denotes lattice oxygen. Due to the energy gap associated with lattice oxygen oxidation, the LOM can break the scaling relationships inherent in the AEM, often leading to higher oxygen evolution activity. However, in acidic systems, the involvement of lattice oxygen facilitates proton attack, resulting in surface structural degradation and catalyst dissolution. Therefore, the LOM is unfavorable for the long-term stability of HEs under acidic conditions.

*Oxide path mechanism (OPM).* The OPM forms the O–O bond through the direct coupling of two *O species on adjacent metal sites. This process similarly bypasses the scaling relations in the AEM, thereby overcoming the overpotential limitation. Moreover, by involving nearby adsorbed *O species rather than lattice oxygen in the O–O coupling steps (Fig. 2h), the catalyst structure remains intact. As a result, OPM is considered to enable HEs to achieve both high activity and excellent stability. It can be described by the following steps (Fig. 2i):


(11) \begin{eqnarray*}
{\mathrm{M}} {-} {\mathrm{M\ }} + {\mathrm{\ }}{{\mathrm{H}}}_2{\mathrm{O\ }}\rightarrow {\mathrm{\ M}} {-} {\mathrm{M}} {-} {\mathrm{OH\ }} + {\mathrm{\ }}{{\mathrm{H}}}^ + {\mathrm{ }} + {\mathrm{ }}{{\mathrm{e}}}^ -,
\end{eqnarray*}

(12) \begin{eqnarray*}
&& {\mathrm{M}} {-} {\mathrm{M}} {-} {\mathrm{OH\ }} + {\mathrm{\ }}{{\mathrm{H}}}_2{\mathrm{O\ }}\rightarrow {\mathrm{\ HO}} {-} {\mathrm{M}} {-} {\mathrm{M}} {-} {\mathrm{O\ }}\\
&&\quad + {\mathrm{\ }}2{{\mathrm{H}}}^ + {\mathrm{ }} + {\mathrm{ }}2{{\mathrm{e}}}^ -,
\end{eqnarray*}

(13) \begin{eqnarray*}
&& {\mathrm{HO}} {-} {\mathrm{M}} {-} {\mathrm{M}} {-} {\mathrm{O\ }}\rightarrow {\mathrm{\ O}} {-} {\mathrm{M}} {-} {\mathrm{M}} {-} {\mathrm{O\ }}\\
&&\quad + {\mathrm{\ }}{{\mathrm{H}}}^ + {\mathrm{\ }} + {\mathrm{\ }}{{\mathrm{e}}}^ -,
\end{eqnarray*}

(14) \begin{eqnarray*}
{\mathrm{O}} {-} {\mathrm{M}} {-} {\mathrm{M}} {-} {\mathrm{O\ }}\rightarrow {\mathrm{\ M}} {-} {\mathrm{M\ }} + {\mathrm{\ }}{{\mathrm{O}}}_2.
\end{eqnarray*}

Extensive efforts have been devoted to developing HEs that promote the OPM pathway. To achieve efficient O–O coupling, HEs of the AOER must not only possess appropriate metal site spacing and geometric configuration, but also ensure the stable adsorption of *O species and suitable reaction energy barriers, and additionally, *in situ* capturing of the evolution of key intermediates, which in turn places higher demands on the atomic-level design of HEs and advanced *in situ* characterization techniques. To this day, the exploration of the OER mechanism continues. With the ongoing advancement of *in situ* characterization techniques, it is foreseeable that the veil over the OER mechanism is gradually being lifted.

### OER mechanism regulations

The diverse reaction mechanisms significantly impact the activity and stability of the AOER process, prompting recent studies to diligently uncover the determinants of the reaction pathway. Here, we summarize recent findings and systematically analyze how various factors influence the transition between different AOER mechanisms. In this section, we consolidate recent findings and conduct a systematic analysis of how various factors affect the transitions among different AOER mechanisms. This understanding is crucial for guiding the rational design of next-generation HEs of the AOER that can achieve both high efficiency and long-term durability. This comprehension is vital for directing the strategic design of next-generation HEs of the AOER, aiming to attain both superior efficiency and sustained durability.

**Figure 3.**
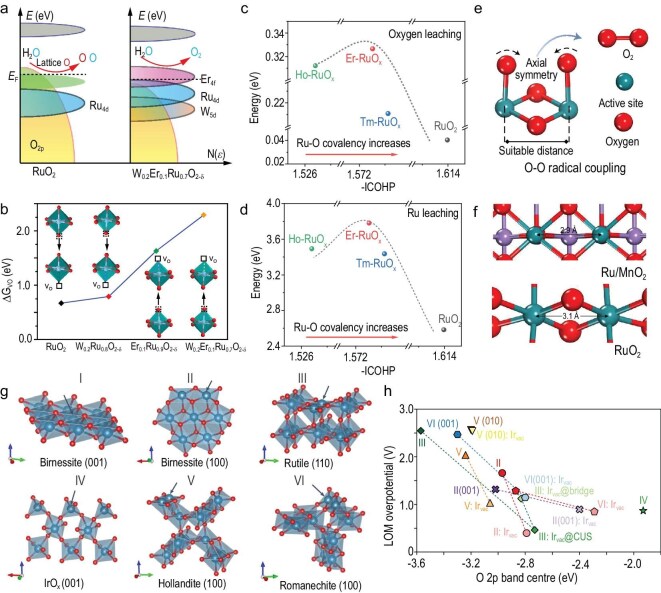
(a) Schematic diagrams of rigid band models for RuO_2_ and W_0.2_Er_0.1_Ru_0.7_O_2−δ_ toward the AOER, and (b) the calculated energy for formation of V_O_ in different positions of RuO_2_, W_0.2_Ru_0.8_O_2−δ_, Er_0.1_Ru_0.9_O_2−δ_ and W_0.2_Er_0.1_Ru_0.7_O_2−δ_. Adapted with permission from Hao *et al.* [28]; Copyright 2020, Springer Nature. (c) The Δ*G*_O vacancy_ and (d) the Δ*G*_Ru vacancy_ as a function of the integrated crystal orbital Hamilton population (ICOHP) for lanthanide-doped RuO_x_. Adapted with permission from Li *et al.* [34]; Copyright 2024, Springer Nature. (e) O–O radical coupling promoted by symmetric dual active sites. (f) Ru–Ru interatomic distances in RuO_2_ and α-MnO_2_ with Ru atoms substituting for the Mn at surface sites. Adapted with permission from Lin *et al.* [35]; Copyright 2021, Springer Nature. (g) Structural models of birnessite (001), birnessite (100), rutile (110), IrO_x_ (001), hollandite (100) and romanechite (100), and (h) corresponding correlation between the computed OER LOM overpotential and the O 2*p* band center for selected motifs. Adapted with permission from Lu *et al.* [40]; Copyright 2024, Springer Nature.

*O* 2*p band center.* The electronic structure is frequently emphasized, as it directly relates to the reactivity of metal sites and lattice oxygen in nanocrystalline HEs of the AOER. Importantly, the O 2*p* band center has been regarded as an appropriate descriptor for evaluating the oxygen vacancy formation energy and surface oxygen exchange kinetics in solid HEs of the AOER [23]. Adjusting the energy gap between the O 2*p* band center and the Fermi level can enhance the reactivity of lattice oxygen, thereby facilitating electron loss during electrochemical oxidation processes. thereby partially activating the reactivity of lattice oxygen and increasing the contribution of the LOM pathway during the OER. It is widely accepted that the O 2*p* band center is a key factor governing the transition between the AEM and LOM, influencing OER activity in perovskite oxides [24], spinel oxides [25,26] and transition metal (oxy)hydroxides [27] under alkaline conditions. Recently, the O 2*p* band center theory has also been applied to explain and guide the design of highly durable RuO_2_ HEs for the AOER. For example, Hao and co-workers doped W and Er into the RuO_2_ lattice, which widened the energy gap between the O 2*p* band center and the Fermi level (Fig. 3a), increased the oxygen vacancy formation energy (Fig. 3b) and significantly enhanced the long-term stability of RuO_2_ [28]. Similarly, Ping and co-workers suppressed the LOM pathway by downshifting the O 2*p* band center, effectively locking the lattice oxygen and greatly reducing its participation in the AOER process [29].

*Metal–oxygen covalency.* In addition to the energy gap between the O 2*p* band center and the Fermi level, the energy gap between the O 2*p* band and the metal (M) *d* band has also been taken into account. M–O hybridization and covalency in the OER mechanism have increasingly garnered the attention of researchers [30–32]. Strong M–O covalency, accompanied by *e_g_* orbital filling, shows a strong correlation with OER activity in perovskite oxides, suggesting that M–O hybridization plays a significant role in influencing the OER process [33]. Grimaud and co-workers proposed that the energy gap between the O 2*p* band and the M *d* band can serve as an indicator of M–O covalency, which plays a crucial role in determining the reactivity of lattice oxygen [24]. A smaller energy gap indicates stronger M–O hybridization and greater covalency. The strong covalent M–O bonds allow metal sites to more readily accept electrons from the energetically proximate O 2*p* orbitals during redox processes. As a result, electron transfer from lattice oxygen to the metal sites is facilitated, increasing the risk of lattice oxygen oxidation and making the LOM pathway more likely to occur. Recently, strategies to regulate M–O covalency have been applied to the widely studied RuO_2_ HEs for the AOER (Fig. 3c and d). Weakened Ru–O covalency can inhibit lattice oxygen participation in the AOER and suppress oxygen vacancy formation. However, excessively weakening the Ru–O covalency may make Ru easily leached [34]. Therefore, moderately weakened Ru–O covalency is considered beneficial for suppressing the LOM pathway and enhancing the durability of RuO_2_ HEs.

*Active sites distance.* The OPM pathway, which enables direct O–O coupling between adjacent adsorbed *O species, not only breaks the activity limitations imposed by the AEM but also avoids the poor stability associated with oxygen vacancy formation in the LOM mechanism. This reaction pathway has been validated in homogeneous iron complexes and subsequently in HEs under alkaline conditions [25,26]. However, despite recent advancements such as the introduction of cationic defects to enhance the synergistic interaction of dual-metal active sites, realizing OPM under acidic conditions continues to be a significant challenge. The prevailing view holds that the core of the OPM mechanism lies in the presence of adjacent active sites. Therefore, regulating the distance between active sites to promote direct O–O coupling is considered a key strategy for increasing the contribution of the OPM pathway. Recently, Lin and co-workers designed and synthesized an arrayed configuration of Ru atoms on α-MnO_2_ [35], wherein the adjacent Ru sites enabled the successful realization of the OPM pathway (Fig. 3e and f). Thereby, both the activity and durability of the AOER are significantly enhanced.

*Local paracrystalline structure* The influence of local structure on the OER mechanism is also attracting growing attention from researchers. Local strain can modulate the spatial distance between atoms, further affecting the degree of orbital hybridization between metal and oxygen (M–O), regulating the electronic overlap between metal *d* orbitals and oxygen *p* orbitals. Such hybridization changes not only influence the adsorption strength of intermediates but can also enhance the reactivity of lattice oxygen, thus promoting the occurrence of the LOM or even OPM pathway [36,37]. Moreover, non-uniform coordination environments, such as low-coordinated metal sites, edge sites and defect regions, can lead to deviations in local electronic structure, breaking conventional linear adsorption energy relationships and offering new possibilities to overcome the limitations of the AEM [38,39]. Recently, surface-reconstructed paracrystalline IrO_x_ has demonstrated outstanding AOER activity, far surpassing that of bulk IrO_2_. Lu and co-workers investigated the paracrystalline structural motifs present on the surface of restructured Ir-based perovskites (Fig. 3g). They found that different types of paracrystalline IrO_x_ contribute to AOER activity and stability through distinct reaction mechanisms (Fig. 3h) [40]. This highlights the critical role of paracrystalline local structures in governing the AOER mechanism.

### HE degradation and failures in the AOER and PEMWEs

**Figure 4.**
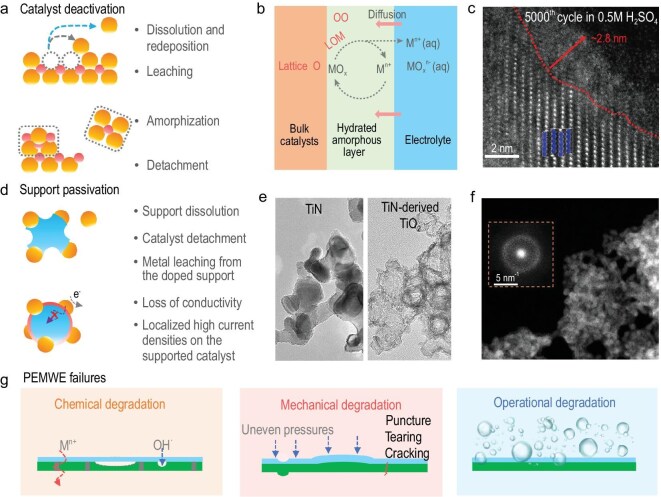
Failures of the catalyst and device for the AOER. (a) Schematic diagrams of AOER catalyst deactivation. (b) Surface oxidation, dissolution and structural reconstruction of metal oxide catalysts in the AOER. (c) Lattice-resolved high-angle annular dark-field scanning transmission electron microscopy (HAADF-STEM) image of the surface structure of pristine Ca_2−_*_x_*IrO_4_ after 5000 CV cycles in 0.5 M H_2_SO_4_. Adapted with permission from Li *et al.* [41]; Copyright 2022, American Chemical Society. (d) Schematic diagrams of support passivation of AOER catalysts. (e) TEM images of pristine TiN and TiN after 100 h at 1.6 V (denoted as TiN-derived TiO_2_) and (f) HAADF-STEM image of Ir/TiN after 100 h test at 10 mA cm^−2^. Inset is the corresponding fast Fourier Transform (FFT) pattern. Adapted with permission from Lin *et al.* [44]; Copyright 2025, Wiley-VCH. (g) PEMWE failure during long-term stability operation.

The practical water electrolysis devices face stability challenges during long-term operation, which are often related to the intrinsic catalytic mechanisms, structural characteristics and actual device assembly processes. Catalyst deactivation is associated with structural instability, including reconstruction, amorphization, leaching, dissolution–redeposition and detachment (Fig. 4a). In acidic media, the attack of highly concentrated protons induces lattice oxygen in bulk metal oxide catalysts to lose electrons, migrate to the surface, and eventually be released via the LOM pathway. This process leads to surface amorphization and the formation of a loose hydrated layer. Furthermore, once the stabilizing role of lattice oxygen in maintaining the oxide framework is lost, partial leaching of metal cations occurs in the form of metal ions (Mn^+^) or oxygen-containing species (MO_x_^n−^), some of which may redeposit while others permanently detach from the catalyst surface (Fig. 4b). Typically, the surface amorphization of Ca_2−x_IrO_4_ nanocrystals can be observed during the acidic OER in H_2_SO_4_ electrolyte (Fig. 4c). This phenomenon originates from lattice oxygen release coupled with Ca^2^⁺ leaching [41]. In RuO_2_ and IrO_2_ lattices, the octahedral coordination gradually transforms into lower-coordination species, such as IrO_3_ (trigonal) [42] and RuO_4_ (tetrahedral) [43], upon lattice oxygen loss. Recent advances have confirmed the formation of RuO_4_ as a dissolution intermediate, highlighting that catalyst deactivation originates from structural collapse induced by the oxidative release of lattice oxygen.

Beyond the deactivation of active sites, many practical AOER catalysts rely on supports to reduce noble-metal loading or to alleviate mass transport limitations. However, support passivation also represents a critical failure mode during long-term operation. The ideal support must balance conductivity with excellent acid resistance, yet some conductive supports suffer from severe corrosion under acidic oxidative conditions (Fig. 4d). For example, in IrO_x_ catalysts supported on TiN (Ir/TiN), the TiN support undergoes acidic oxidative reconstruction into hollow TiO_x_ shells (Fig. 4e and f) [44]. Stabilizing supports to prevent corrosion-induced detachment of catalytic centers has gradually emerged as an effective strategy for constructing robust AOER catalysts. For instance, Mo doping suppresses the dissolution of MnWO_4_ supports, enabling Ir nanoparticles supported on MnW_0.95_Mo_0.05_O_4_ to retain both their morphology and nanoparticle dispersion even after acidic oxidative testing [45]. In addition to corrosion-induced catalyst detachment, some conductive supports suffer from a decline in intrinsic conductivity due to the leaching of dopant metals. A classic example is Sb-doped SnO_2_ (antimony-doped tin oxide) (IrO_x_/ATO), where the dissolution of Sb has been observed during long-term AOER testing, leading to the degradation of the support conductivity [46]. Low conductivity of the support can lead to locally elevated current densities on IrO_x_ nanoclusters. This not only compromises the structural stability of the IrO_x_ clusters but also causes severe voltage fluctuations due to poor electrical contact.

The practical application of catalysts in PEMWEs is mainly challenged by issues associated with the membrane electrode assembly (MEA). Both the fabrication process and the operating conditions of the MEA significantly affect the power output and long-term durability of PEMWEs. The failure of PEMWEs is generally categorized into chemical degradation, mechanical degradation and operational degradation (Fig. 4g). As the most widely used PEM on the market, Nafion exhibits proton conductivity that relies on water-filled transport channels, which restricts the operating temperature to between 0°C and 100°C. Moreover, the feed water in PEMWEs often contains trace metal ions (e.g. Ca^2^⁺, Mg^2^⁺) or dissolved catalyst species. These ions can occupy the transport channels of the PEM, and subsequently migrate and deposit on the cathode side, thereby introducing additional ohmic resistance [47,48]. In addition, oxygen leaking to the cathode side may undergo incomplete reduction, leading to the formation of reactive free radical species (·HO, ·HO_2_) [49,50]. These oxidative radicals can attack the PEM and the catalytic layer, accelerating chemical degradation and compromising the long-term durability of the PEMWE. Mechanical degradation results from the mechanical failure of the PEM. High-pressure operation in PEMWEs can cause puncture, tearing or cracking of the membrane. In addition, non-uniform pressure applied during MEA fabrication may lead to uneven catalyst layers, resulting in localized high current densities, which impair mass transport and exacerbate the damage to the catalyst layer. The stability of PEMWEs is also strongly influenced by operating conditions, such as gas bubble formation, operating temperature and water supply. At high current densities, gas bubble accumulation on the catalyst surface can block active sites and hinder mass transport. The flow-field design of PEMWEs, together with the water supply method and flow rate, also plays a crucial role in regulating mass transport. Poor water management not only exacerbates bubble-induced transport limitations but may also cause local drying or flooding within the membrane electrode assembly, leading to uneven current distribution and accelerated performance degradation.

## RECENT ADVANCES IN DESIGN STRATEGIES OF HES FOR THE AOER

HEs for the AOER aim to achieve the dual goals of high activity and high stability. Beyond a fundamental understanding of the OER mechanism and influencing factors, the practical design strategies for different types of HEs, primarily Ir-based, Ru-based and non-precious-metal HEs such as Mn-based and Co-based systems, have also become a central focus. reducing Ir loading while enhancing its intrinsic activity, in order to lower the manufacturing cost of PEMWEs and improve overall energy efficiency, ultimately facilitating the industrial implementation of PEMWEs.

Current research focuses on improving their durability to make them low-cost HEs of the AOER as potential substitutes for Ir-based materials. Meanwhile, the development of Mn- and Co-based HEs involves stabilizing their metastable active phases and enhancing their acid resistance. Although highly challenging, ongoing research on non-precious-metal-based HEs of the AOER remains inspiring and full of promise. Herein, we provide an overview of recent research progress on Ir-based, Ru-based, Mn-based and Co-based HEs and summarize representative strategies for catalyst design. These tailored design approaches, guided by mechanistic insights, are paving the way toward next-generation HEs of the OER for practical application in acidic environments.

### Ir-based HEs of the AOER

Currently, most industrial HEs of the AOER are Ir-based. However, the extremely high cost (∼US$60 670 per kg) and substantial loading requirements (>0.5 mg_Ir_ cm^−^^2^) pose significant challenges for practical electrochemical devices. In addition, the energy efficiency targets of PEMWEs (such as the 2025 U.S. Department of Energy (DOE) goal of <1.9 V at 3 A cm^−^^2^) demand further improvements in the AOER activity of commercial Ir-based HEs. Therefore, reducing Ir loading while enhancing AOER activity has become the primary design objective for Ir-based HEs.

Ultrasmall IrO_x_ nanocrystals can exhibit exceptionally high mass activity, but they typically require integration with high-surface-area, acid-resistant supports. Benefiting from strong metal–support interactions, supported Ir HEs often show modulated electronic structures and optimized adsorption energies for oxygen intermediates, resulting in superior AOER performance compared to pure IrO_x_ [51]. The conductivity, acid resistance and oxidative stability of the support are carefully considered. For example, in the IrO_x_/Sb:SnO_2_ (Ir/ATO) system, dopants with different oxidation states affect the conductivity of the ATO support. Poorly conductive ATO hinders electron transfer between the support and IrO_x_, thereby reducing the AOER activity of the Ir/ATO catalyst [52]. The stability of the support also plays a key role in determining catalyst durability.

**Figure 5.**
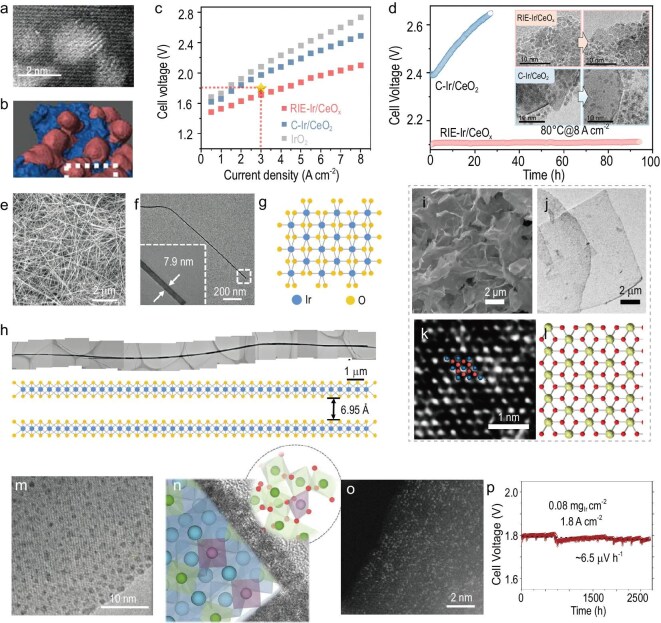
(a) Spherical aberration-corrected HAADF-STEM image and (b) 3D reconstructed model of CeO_x_ of Ir loading. (c) Polarization curves of PEMWEs and (d) chronopotentiometric curves of PEMWEs using RIE-Ir/CeO_x_ and C-Ir/CeO_2_ operated at 8 A cm^−2^. The inset shows TEM images of the catalysts taken before and after operation. (a–d) Adapted with permission from Shi *et al.* [54]; Copyright 2025, American Association for the Advancement of Science. (e) Scanning electron microscopy (SEM) image, (f) TEM image and (g) pictorial illustration of the crystal structure of IrO_2_ nanoribbons. (h) A typical long IrO_2_ nanoribbon with a length of 22.53 μm. Adapted with permission from Liao *et al.* [58]; Copyright 2023, Springer Nature. (i) SEM image, (j) TEM image, (k) HAADF-STEM image and (l) the atom model of 1T-IrO_2_. Adapted with permission from Dang *et al.* [56]; Copyright 2021, Springer Nature. (m) High-resolution TEM (HRTEM) image of Pr_3_IrO_7_ at electrochemical cycles. (n) Schematic illustrations of the catalyst structure after reconstruction. Adapted with permission from Chen *et al.* [68]; Copyright 2023, Springer Nature. (o) HAADF-STEM image of Ir^VI^–MnO_2_ and (p) cell voltages of the MEA assembled by Ir^VI^–MnO_2_ measured at 1.8 A cm^−2^. Adapted with permission from Li *et al.* [72]; Copyright 2024, American Association for the Advancement of Science.

On unstable supports or those with weak interactions with Ir species, the active Ir species are prone to detachment or aggregation during the AOER process [53]. Recently, Shi and co-workers developed a ripening-induced embedding (RIE) strategy to securely embed IrO_x_ into metal oxide supports (Fig. 5a and b) [54]. This embedded architecture significantly enhances the metal–support interaction, effectively preventing IrO_x_ aggregation and detachment while maintaining excellent catalytic activity. As a result, the assembled PEMWE device required only 0.4 mg cm^−^^2^ of total platinum group metal (PGM) loading (for both the anode and cathode) and achieved a cell voltage of 1.72 V at a current density of 3 A cm^−^^2^ and ultra-low performance degradation at industrial current density of 8 A cm^−^^2^ (Fig. 5c and d). For supported IrO_x_ systems, the intrinsic activity of IrO_x_, the stability and electronic structure of the support, as well as the metal–support interaction, have become key focal points for the future design of Ir-based HEs.

Low-dimensional design and crystal structure engineering also represent practically meaningful strategies to reduce Ir loading and enhance the intrinsic activity of Ir-based HEs. Low-dimensional structures (such as 1D nanowires and 2D nanosheets) not only expose more active sites due to their high surface-to-volume ratio, but also facilitate charge transport and mass diffusion during the AOER process. Moreover, the structural anisotropy and surface unsaturation in these low-dimensional materials can modulate the local electronic environment of Ir sites, thereby optimizing the adsorption energies of oxygenated intermediates and promoting reaction kinetics. The stable rutile phase of IrO_2_ poses challenges for its oriented growth into 1D or 2D structures. In contrast, metastable phases of IrO_2_ naturally exhibit 1D or 2D morphologies and have been gaining increasing attention.

Rutile IrO_2_ can be transformed into layered A_x_Ir_y_O_z_ compounds via the intercalation of alkali metal ions (A⁺) and subsequent phase transformation [55,56]. These layered intermediates can then be exfoliated into 2D IrO_2_ nanosheets with a hexagonal lattice through acid treatment and cation exchange (e.g. with tetrabutylammonium ions). By further optimizing this synthetic strategy, such as selecting different Ir precursors, e.g. IrO_2_, IrCl_3_ or Ir(acac)_3_, varying the alkali metal sources (e.g. alkali metal carbonates or hydroxides) and tuning the reaction temperature, various metastable IrO_2_ phases have been successfully obtained, including 1T-IrO_2_ (Fig. 5e–h) [56], 3R-IrO_2_ [57], monoclinic IrO_2_ (Fig. 5i–l) [58] and amorphous IrO_x_ nanosheets [59]. These low-dimensional Ir-based structures exhibit significantly enhanced intrinsic activity compared to bulk IrO_2_. Their implementation in MEAs enables low loading while maximizing Ir utilization. Moreover, their favorable porous architectures facilitate efficient mass transport, including the release of oxygen bubbles and the delivery of water [60]. Additionally, the metastable structures offer valuable insights into the relationship between structural motifs and AOER activity, shedding light on how atomic arrangement influences catalytic performance.

Extensive studies have been conducted on surface reconstruction in Ir-based HEs. In perovskite oxides [61–66] and spinel oxides [67], Ir species tend to reconstruct into ultrasmall IrO_x_ nanoparticles under acidic conditions and during the AOER, typically through the leaching of A-site or B-site cations. These nanoparticles often exhibit significantly enhanced AOER activity (Fig. 5m and n) [68]. Recent research has focused on uncovering the reasons behind the improved activity of reconstructed IrO_x_ nanoparticles. Zhao and co-workers, through studies on the surface reconstruction of Co-doped SrIrO_3_, attributed the activity enhancement to the formation of low-coordination IrO_x_ structures [69]. Lu and co-workers combined X-ray and electron scattering techniques to reveal three paracrystalline structural motifs at the reconstructed surfaces of SrIrO_3_ perovskite. Several types of short-range ordered Ir–O_6_ octahedral connections were found to contribute to high activity via both the AEM and LOM [40].

Beyond the fundamental understanding of activity origins, controlled reconstruction has also been emphasized due to its critical role in determining catalyst stability. For instance, Ir–O–Mn configurations tend to activate the LOM pathway [70]. The Ir species in the Co_2_MnO_4_ spinel lattice can undergo a phase transformation and controlled reconstruction into highly active IrO_x_/MnO_x_ through a pre-acid treatment under non-electrochemical conditions [67]. Controlled reconstruction to form IrO_x_ with high intrinsic activity offers valuable insights for the development of advanced Ir-based HEs of the AOER.

Atomically dispersed Ir HEs typically incorporate Ir species at the atomic level into the lattice of supporting materials, often metal oxides. The unique coordination environments at these isolated Ir sites confer distinctive AOER activity and significantly improve Ir utilization. In supports that are intrinsically inert toward the AOER, the density of atomic Ir sites plays a major role in determining the apparent catalytic activity. For example, Li and co-workers integrated interactive Ir atoms into the titanium oxide lattice (i-Ir/TiO_2_) [71]. Although i-Ir/TiO_2_ exhibits higher mass activity than Ir nanoparticles, the enhancement of apparent activity still relies on achieving a high density of atomic Ir sites. In contrast, when supported on catalytically active hosts, the synergy between atomic Ir and the support enables exceptional AOER performance.

Li and co-workers reported that atomically dispersed high-valent Ir in MnO_2_ (Ir^VI^–MnO_2_, Fig. 5o) delivered an impressive mass activity of 1.7 × 10^5^ A g_Ir_^−1^ and outstanding durability, maintaining operation at a current density of 1.8 A cm^−2^ for 2700 h in a PEMWE device (Fig. 5p) [72]. However, in less stable supports such as MnO_2_ and Co_2_MnO_4_ [67,70], atomically dispersed Ir species tend to reconstruct into aggregated IrO_x_ nanoparticles during electrochemical operation. It remains unclear whether the final stable phase is truly atomic Ir species or reconstructed IrO_x_, and further investigation is still required.

### Ru-based HEs of the AOER

Ru-based HEs are considered the most promising alternatives to Ir-based HEs for the AOER, owing to their outstanding intrinsic activity and significantly lower cost, ranging from only one-fifth to one-sixteenth of that of Ir [73,74]. Despite these advantages, the practical deployment of Ru-based HEs remains severely limited by their poor electrochemical stability. Growing research efforts have been directed toward improving their stability, particularly under industrially relevant high current densities [75–78].

**Figure 6.**
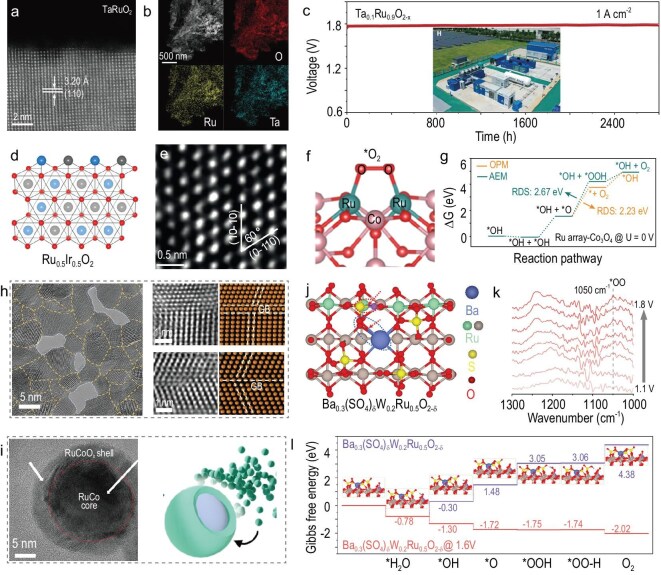
(a) Cross-sectional HAADF-STEM image and (b) energy-dispersive spectroscopy (EDS) mapping of Ta_0.1_Ru_0.9_O_2−x_. (c) Voltage–time curves of PEMWEs with Ta_0.1_Ru_0.9_O_2-x_. Adapted with permission from Zhang *et al.* [82]; Copyright 2025, American Association for the Advancement of Science. (d) Atomistic structure and (e) high-magnification aberration-corrected HAADF-STEM image of Ru_0.5_I_r0.5_O_2_. Adapted with permission from Zhu *et al.* [85]; Copyright 2023, Springer Nature. (f) Schematic illustration of the key intermediates during the OPM pathways and (g) Δ*G* diagrams for the OER via the OPM and AEM pathways over Ru array–Co_3_O_4_. Adapted with permission from Chang *et al.* [91]; Copyright 2024, American Chemical Society. (h) HR-TEM image, high-magnification images and simulated structure of the GB of GB–RuO_2_. Adapted with permission from He *et al.* [96]; Copyright 2024, Wiley-VCH. (i) HRTEM image and schematic illustration of Co–Ru@RuO_2_. Adapted with permission from Chen *et al.* [100]; Copyright 2025, American Chemical Society. (j) Ball-and-stick model of Ba-anchored sulfate on the RuO_2_ (110) plane. (k) *In situ* ATR-SEIRAS spectra and (l) Gibbs free energy diagrams on the RuO_2_ (110) plane of Ba_0.3_(SO_4_)*_δ_*W_0.2_Ru_0.5_O_2−δ_. Adapted with permission from Xue *et al.* [104]; Copyright 2023, Springer Nature.

Doping has emerged as one of the most widely studied modification strategies for Ru-based HEs [79–81], as it can enhance catalyst stability without compromising the apparent AOER activity of RuO_2_. A growing number of works have demonstrated that doping can effectively tune the electronic structure of RuO_2_, modulate the dynamic changes in Ru oxidation states during the AOER, accelerate proton-coupled electron transfer steps and optimize the adsorption energies of key OER intermediates. Zhang and co-workers achieved enhanced intrinsic AOER activity and suppressed corrosion of RuO_2_ through Ta doping (Fig. 6a and b) [82]. Ta incorporation effectively suppressed the transformation of Ru sites into unstable high-valence intermediates during the AOER, significantly reducing Ru dissolution while mitigating surface degradation through inhibited cluster exsolution and reconstruction.

In PEMWE evaluations, the Ta–RuO_2_ electrocatalyst exhibited a remarkably low performance degradation rate of ∼14 μV h^−^^1^ over 2800 h of continuous operation at an industrial current density of 1 A cm^−2^ (Fig. 6c). Deng and co-workers proposed a strategy by introducing corrosion-resistant p-block metal elements into the RuO_2_ matrix to achieve bond length modulation between Ru active sites and dopants (Fig. 6d) [83], thereby optimizing proton transport capability. This approach resulted in highly asymmetric Ru–O–M moieties with strong electronic coupling effects, accelerating the deprotonation process during the AOER.

Integrating highly active Ir atoms into RuO_2_ matrices to steer reaction pathways away from lattice oxygen participation was reported recently [73,84]. This approach maximizes synergistic Ru–Ir interactions through lattice parameter-modulated templating of their variable growth behavior, effectively balancing AOER activity and structural stability. Zhu and co-workers developed a stable 2D Ru_0.5_Ir_0.5_O_2_ oxide with oxidized surface charges as an efficient AOER catalyst (Fig. 6e) [85]. Its enhanced activity originates from Ir-induced formation of Ru sites with elevated oxidation states at low potentials and increased surface charge density during the AOER. The Ru_0.5_Ir_0.5_O_2_ catalyst demonstrated robust stability under 10 mA cm^−2^ operation, maintaining performance over 618 h. Similarly, a unique Ru@Ir core–shell electrocatalyst was developed through sequential deposition of Ru (core) and Ir (shell) atoms on lattice-expanded Ni_3_S_4_ by Park and co-workers [86]. This (RuIr)O_2_/C configuration enhances stability and facilitates AOER kinetics via optimized interfacial electronic interactions. Bertelsen and co-workers synthesized a series of Ir_1−x_Ru_x_O_2_ solid-solution nanoparticles via hydrothermal synthesis and revealed the formation of metal–oxygen octahedral clusters during synthesis and their role in the crystal structure [87]. Their research also proved that introducing trace Ir can significantly enhance the AOER performance of RuO_2_ by achieving the optimal balance between catalytic activity and stability.

The OPM bypasses conventional limitations by enabling direct O–O radical coupling, avoiding both high *OOH formation energy in the AEM and structural instability from lattice oxygen release in the LOM. This mechanism provides a promising solution to the activity–stability trade-off of the AOER for RuO_2_ in an acidic environment [81,88,89]. Optimal metal–metal distances are critical for OPM initiation, requiring strategic metal site incorporation in RuO_2_ to precisely modulate metal–metal bond lengths for OPM activation. Recently, integrating atomic arrays of Ru into the surface of oxide supports (e.g. MnO_2_, Co_3_O_4_) has enabled the formation of adjacent Ru atoms with shorter interatomic distances compared to those on the surfaces of RuO_2_ [90,91].

The closer proximity of Ru atoms facilitates O–O coupling, making it more favorable during the AOER process (Fig. 6f and g). Additionally, enhancing OH coverage via oxygen-affinitive atom incorporation to enable the OPM pathway has also been reported. Wang and co-workers proposed a spatiotemporal oxygen radical regulation strategy by incorporating single-atom Ga sites into interconnected RuO_2_ nanocrystal frameworks (Ga_x_Ru_1−x_O_2_), forming heterogeneous donor-reaction site pairs that enable direct radical coupling to activate the OPM [92]. Cao and co-workers developed a Cr_0.6_Ru_0.4_O_2_ solid-solution electrocatalyst by incorporating Lewis acidic Cr sites into RuO_2_ [93]. Cr accelerated water dissociation, promoted OH adsorption and optimized dual-site spacing to facilitate *O radical coupling, shifting the AOER mechanism to a Cr–Ru dual-site OPM.

Grain boundaries (GBs) can induce structural defects, including vacancies, dislocations and bond distortions, creating strained atomic coordination environments that modulate the electronic structures of active sites [94]. Densely packed GBs generate unsaturated coordination, tensile stresses and high-energy surfaces that can not only regulate electronic configurations and intermediate adsorption energies but also provide anti-relaxation effects for improved AOER kinetics and stability [95]. A GB engineering strategy shows potential to resolve the persistent activity–stability trade-off in RuO_2_-based HEs.

He and co-workers fabricated a GB-engineered RuO_2_ catalyst (GB-RuO_2_) as ultrathin porous nanosheets (Fig. 6h) [96]. GBs induced tensile stress and unsaturated coordination, optimizing intermediate adsorption and stabilizing active sites during the AOER. *Operando* characterization confirmed enhanced stability through suppressed Ru dissolution and an inhibited LOM path. Wu and co-workers engineer V-doped RuO_2_ with high-density microcrystalline GBs without templating agents [97]. Atomic-level characterization revealed V/GB co-modulation precisely adjusts Ru–O bonds, generating low-valence/low-coordination Ru sites while weakening Ru–O hybridization, which suppresses the LOM and maintains AEM dominance across AOER potentials for efficient high-current-density operation.

Interface engineering can enhance electrocatalytic activity in hybrid nanomaterials through synergistic dual/multi-active site effects [98]. Rational heterostructure design enables spatially proximate reaction steps across distinct active components, accelerating overall kinetics. This coupling further modulates electronic states, as exemplified by Mott–Schottky effects at Janus metal–semiconductor interfaces, facilitating rapid multi-electron transfer [99]. Recently, the core–shell structure of Ru@RuO_2_ (CoRu@RuO_2_) HEs with integrated atoms has been developed (Fig. 6i).

The metal–metal oxide core–shell architecture enhances the stability of Ru-based HEs, achieving a low overpotential of 203 mV and excellent stability over a 400-h durability test at 10 mA cm^−2^ [100]. Liu and co-workers developed a core–shell RuCo/RuCoO_x_ structure with confined Schottky heterojunctions via controlled oxidation of RuCo alloys [101]. This design maximizes active site accessibility while enhancing durability and intrinsic AOER activity. Schottky-induced lattice strain and charge transfer modulate electronic structures, suppressing Ru over-oxidation/dissolution and protecting the metallic RuCo core from acid corrosion. Lu and co-workers reported a controlled metallic Ru deposition strategy on RuO_2_ surfaces to construct Ru^4+^–O–Ru^0^ interfaces [102]. Metallic Ru acts as an electron donor, reducing *V_O_–RuO_4_^2−^ oxidation states in Ru/RuO_2_ and stabilizing its structure for enhanced AOER stability. Concurrently, Ru^4+^–Ru^0^ interfacial oxygen sites accelerate deprotonation kinetics, boosting AOER activity.

The anion doping/modification has been reported as an effective approach to break *OH/*OOH scaling relations, modulate Ru oxidation states and stabilize lattice oxygen [103]. Anion-modified RuO_2_ reduces intermediate formation energy while stabilizing Ru sites and lattice oxygen, synergistically boosting AOER activity and durability. Xue and co-workers developed an oxygen-containing anion (SO_4_^2−^) protection strategy, where coordinatively unsaturated O atoms bond with Ru sites on RuO_2_ surfaces to stabilize lattice oxygen (Fig. 6j) [104]. *In situ* ATR-SEIRAS spectra and density functional theory (DFT) calculations reveal that SO_4_^2−^ anions promote the deprotonation of OO–H, thereby accelerating the AOER (Fig. 6k and l). Meanwhile, the Ba_0.3_(SO_4_)_δ_W_0.2_Ru_0.5_O_2−δ_ catalyst-assembled PEMWEs operated stably for 300 h at a current density of 500 mA cm^−2^. Lu and co-workers reported an anion engineering strategy stabilizing sulfate anions on RuO_2_ surfaces via MoO_3_ to design a sulfate-anchored RuO_2_/MoO_3_ multicomponent catalyst. The MoO_3_-mediated sulfate stabilization suppresses leaching during the AOER, achieving enhanced AOER activity and durability.

### Non-noble-metal HEs of the AOER

Low-cost and Earth-abundant non-noble-metal HEs have demonstrated intrinsic AOER activity but suffer the oxidation, dissolution and redeposition of active components, leading to a notable degradation in performance. Notably, non-noble-metal HEs with the potential to maintain both activity and stability under AOER conditions are primarily focused on Mn-based and Co-based oxide materials. However, their stability remains significantly inferior to that of noble-metal-based HEs.

**Figure 7.**
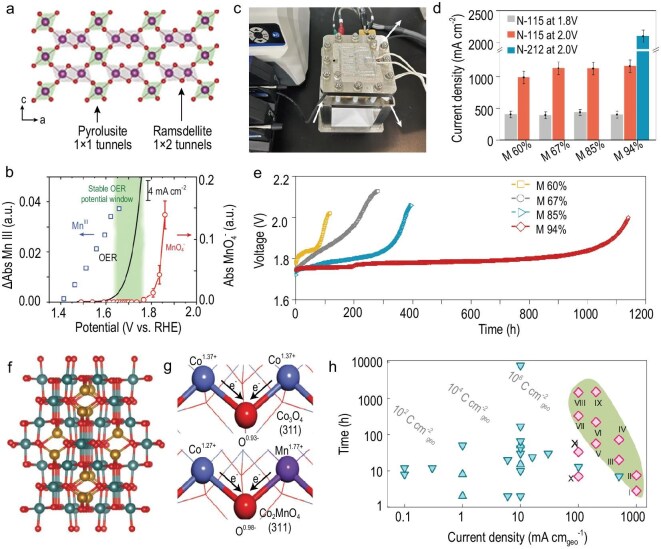
(a) Schematic diagram of γ-MnO_2_ showing an intergrowth structure of pyrolusite and ramsdellite matrices. (b) Potential dependence of Mn^3+^ accumulation (blue squares), the oxygen evolution current (black line) and MnO^4−^ generation (red circles). Adapted with permission from Li *et al.* [105]; Copyright 2019, Wiley-VCH. (c) The set-up of the PEM electrolyzer and (d) current densities of M 60%, M 67%, M 85% and M94% achieved at cell voltages of 1.8 and 2.0 V with N-115 and N-212 membranes, respectively. (e) Long-term durability of M 60%, M 67%, M 85% and M 94% during PEM electrolysis at 200 mA cm^−2^ with N-115 membrane. (c–e) Adapted with permission from Kong *et al.* [106]; Copyright 2024, Springer Nature. (f) Crystal structure of Co_2_MnO_4_. (g) Oxidation state of Co_3_O_4_ and Co_2_MnO_4_ described by differences in electron transfer based on Bader charge calculations. (h) Comparison of the stability of Co_2_MnO_4_ with Co_3_O_4_ and other Earth-abundant OER catalysts. Adapted with permission from Li *et al.* [107]; Copyright 2022, Springer Nature.

Among various manganese dioxide polymorphs, γ-MnO_2_ has attracted considerable attention due to its unique intergrowth structure, consisting of both ramsdellite (R-MnO_2_) and pyrolusite (β-MnO_2_) phases. This intergrowth leads to an alternating arrangement of asymmetric (ramsdellite-type) and symmetric (pyrolusite-type) [MnO_6_] octahedral units, forming a composite framework of single and double chains (Fig. 7a). Such a mixed-phase structure not only enhances the structural rigidity of MnO_2_ but also improves its chemical resilience in acidic environments.

Notably, the presence of robust Mn–O bonding and dense framework connectivity in ramsdellite domains contributes significantly to the suppression of Mn dissolution and structural degradation under AOER conditions. γ-MnO_2_ HEs, by depositing Mn precursors on carbon paper, exhibited a low overpotential of 420 mV and long-term stability over 8000 h at a current density of 10 mA cm^−2^ [105]. However, γ-MnO_2_ can only operate stably at low overpotentials. When the applied potential increases to approximately 1.75 V versus the reversible hydrogen electrode (RHE), the dissoluble MnO_4_^−^ species is observed, resulting in the rapid deactivation of the γ-MnO_2_ catalyst within 100 h (Fig. 7b).

These results demonstrated the existence of a stable potential window between 1.6 and 1.75 V where the AOER can be sustainably catalyzed by γ-MnO_2_. When considering PEMWEs, the stable current must be increased by expanding the stable potential window of γ-MnO_2_. This challenge is addressed by atomically regulating the oxygen structure (planar oxygen and pyramidal oxygen) in the lattice of γ-MnO_2_. By increasing the concentration of planar oxygen (O_pla_) from 60% (M 60%) to 94% (M 94%) within the lattice, γ-MnO_2_ maintained a current density of 200 mA cm^−2^ at 80°C for over 1000 h (Fig. 7c–e) [106]. This 10-fold increase in the catalytic lifetime of γ-MnO_2_ highlights the validity of an extended stable potential window via lattice oxygen regulation for long-term PEM electrolysis.

Recently, Co_2_MnO_4_, which exhibits an activation energy for the AOER comparable to that of state-of-the-art IrO_2_, has been developed by incorporating Mn into the Co_3_O_4_ spinel lattice (Fig. 7f) [107]. Even if several Co atoms are leaching from the Co_2_MnO_4_, the Mn-rich surfaces still exhibit optimal binding energies. Encouragingly, benefiting from a stronger Mn–O bond and the high thermodynamic stability of lattice oxygen (Fig. 7g), Co_2_MnO_4_ HEs can work for over 2 months at 200 mA cm^−2^ at pH 1 without decreasing activity (Fig. 7h).

Co-based HEs have also emerged as promising non-noble-metal candidates for AOER catalysis. For example, Co_3_O_4_ is known to be more active than MnO_2_ under acidic conditions, but Co_3_O_4_ is unstable and dissolves in the form of Co^2+^, even at open circuit potential. Previous studies have also revealed that the deactivation of Co_3_O_4_ results from the formation of undercoordinated CoO sites, which subsequently react with water to form an amorphous 3D porous network of CoO(OH)_x_ layers [108].

**Figure 8.**
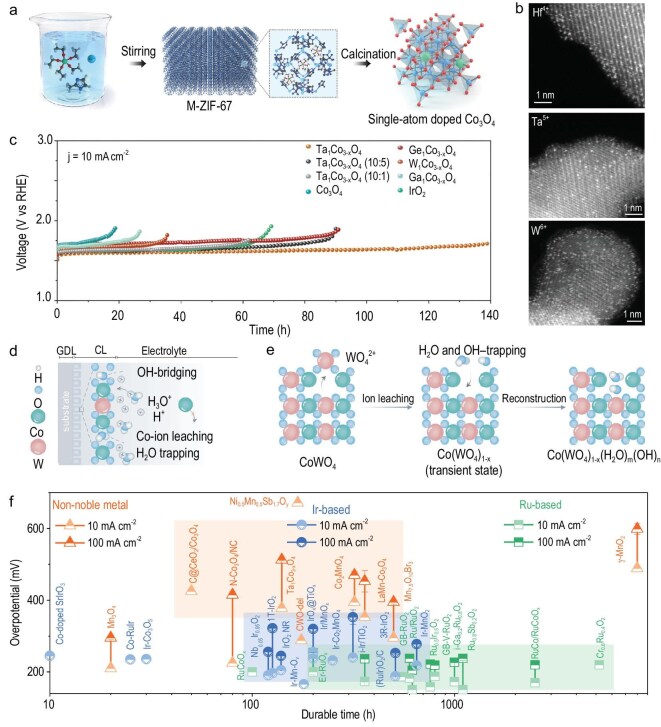
(a) Schematic illustration of the synthetic process of single-atom-doped Co_3_O_4_. (b) HAADF-STEM images of Hf_1_Co_3−x_O_4_, Ta_1_Co_3−x_O_4_ and W_1_Co_3−x_O_4_. (c) Chronopotentiometry curves of the synthesized catalysts and IrO_2_ at 10 mA cm^−2^. Adapted with permission from Lee *et al.* [109]; Copyright 2024, Royal Society of Chemistry. (d) Schematic illustration of the dissolution of Co ions in Co_3_O_4_ through Co–O bond cleaving, followed by their hydration in an acidic medium. (e) Depiction of delaminated Co catalysts (CWO-del-48), illustrating the effect of water trapping and hydroxide bridging within the crystal lattice, enabling stability in acid. Adapted with permission from Ram *et al.* [110]; Copyright 2024, American Association for the Advancement of Science. (f) Comparison map of overpotentials (at 10 and 100 mA cm^−^²) and stability of typical Ir-, Ru- and non-metal-based HEs from Table 1.

Incorporating atomic-scale metal serves as both a catalytically active and protective shell, and has significant implications for enhancing the stability of non-precious-metal HEs that are prone to dissolution under acidic conditions. However, the understanding of the physicochemical characteristics in a diverse range of metal cations is limited, leading to the challenging process of identifying and effectively incorporating suitable cations for substitution in Co spinel oxides (CSOs). Lee and co-workers developed a versatile encapsulation method by confining metal ethoxide as dopant precursor within the pores of ZIF-67 to elucidate the effects of atomic sites, including Hf, Ta, W, Ti, Pd, Ga and Ge, on the surface of the CSO (Fig. 8a) [109]. Such an encapsulation strategy preserves the precursor in its single atom and prevents the generation of hetero-metal oxide byproducts, as shown in Fig. 8b. Ta, W and Ge exhibit greater stability at the octahedral sites at the surface of the CSO rather than within the core, resulting in a high density of Co^2+^ species on the surface. Among these doped metals, Ta dopants display a remarkable AOER overpotential of 378 mV at 10 mA cm^−2^ and a low degradation for the AOER over 140 h (Fig. 8c).

To further adapt for PEMWE anode applications, Ram and co-workers recently demonstrated enhanced control over the AOER mechanism involving only *O and *OH as intermediates by proposing a structural delamination strategy, whereby high-valence sacrificial tungsten (W) elements in a CoWO_4_ (CWO) crystal structure are selectively removed through a water–hydroxide–WO_4_^2−^ anion exchange process (Fig. 8d and e) [110]. This structural delamination provides a Co oxide defect network to trap and stabilize water and hydroxide species. As a result, the Co ion dissolution in acid shows a marked decrease in CWO compared with Co_3_O_4_ due to the water–hydroxide shielding effect. Encouraged by the stabilization of oxide and water–hydroxide networks, the CWO lattice-delaminated (CWO-del) HEs exhibit excellent cell performance in PEMWE systems under industrial operational settings, achieving a current density of 1.8 A cm^−2^ at 2 V and stable operation for 608 h at 1 A cm^−2^ with a cell voltage of 1.77 V. Therefore, we can foresee that incorporating stable foreign elements in Co_3_O_4_ serves as an effective method to increase stability and activity simultaneously, providing opportunities for Co_3_O_4_ to replace Ir in electrochemical industries. Further progress in balancing the activity and stability is essential to realize the full potential of Earth-abundant elements and mitigate the reliance on Ir for PEM electrolysis.

The catalytic performances of typical Ir-, Ru- and non-metal-based HEs for the AOER over the past 3 years are summarized in Table 1 and Fig. 8f. Noble-metal Ru-based catalysts exhibit competitive overpotentials at both low and high current densities, and recent advances have significantly extended their durability, making them promising and less expensive AOER candidates for replacing Ir-based catalysts. However, compared with the industrial requirement of tens of thousands of hours of stable operation in PEMWEs, Ru-based catalysts still require further improvement. Catalysts based on Ir, as a noble metal, combine both high activity and durability. Current design strategies are focused on reducing Ir loading without sacrificing catalytic performance and stability, which could substantially lower the manufacturing cost of PEMWE stacks. Non-noble-metal-based HEs generally exhibit higher overpotentials than their noble-metal counterparts. Their lower Tafel slopes further lead to larger overpotentials under high current densities. Nevertheless, their low cost and Earth-abundant nature make them advantageous for use at high loadings. Although some non-noble-metal HEs can maintain relative stability within a limited low-current window, their poor long-term durability still restricts their application in PEMWEs. Looking ahead, the roadmap for further enhancing the advantages of PEMWEs will center on the development of non-noble-metal HEs, enabling stack manufacturing without reliance on Earth-scarce and costly noble-metal-based catalysts.

**Table 1.** Catalytic performances of typical Ir-, Ru- and non-metal-based HEs for the AOER over the past 3 years.

		Overpotential at	Durable time at	
HEs	Electrolyte	10/100 mA cm^−2^ (mV)	10 mA cm^−2^ (h)	References
1T-IrO_2_	1.0 M HClO_4_	197 (*η*_10_) 322 ± 5 (*η*_100_)	126 (250 mA cm^−2^)	[56]
3R-IrO_2_	1.0 M HClO_4_	188 (*η*_10_) 253 ± 4 (*η*_100_)	511	[57]
IrO_2_ NR	0.5 M H_2_SO_4_	205 (*η*_10_) 245 ± 10 (*η*_100_)	139	[58]
IrO_x_@TiO_x_	0.5 M H_2_SO_4_	255 (*η*_10_) 320 ± 8 (*η*_100_)	200	[60]
Ir-doped CoMn_2_O_4_	0.05 M H_2_SO_4_	232 (*η*_10_) N/A (*η*_100_)	250	[67]
Co-doped SrIrO_3_	0.5 M H_2_SO_4_	245 (*η*_10_) N/A (*η*_100_)	10 (200 mA cm^−2^)	[69]
Ir-MnO_2_	0.5 M H_2_SO_4_	218 (*η*_10_) 278 ± 10 (*η*_100_)	650	[70]
i-Ir/TiO_2_	0.1 M HClO_4_	240 (*η*_10_) 353 ± 20 (*η*_100_)	315 (100 mA cm^−2^)	[71]
Ir-Mn-O_v_	0.1 M HClO_4_	166 (*η*_10_) N/A (*η*_100_)	180 (100 mA cm^−2^)	[139]
Ir-Co_3_O_4_	0.5 M H_2_SO_4_	236 (*η*_10_) N/A (*η*_100_)	30	[140]
Nb_0.05_Ir_0.95_O_2_	0.5 M H_2_SO_4_	191 (*η*_10_) 256 ± 8 (*η*_100_)	120	[141]
Ir/MnO_x_	0.1 M HClO_4_	238 (*η*_10_) N/A (*η*_100_)	200	[142]
Co-RuIr	0.1 M HClO_4_	235 (*η*_10_) N/A (*η*_100_)	25	[143]
RuCoO_x_	1.0 M HClO_4_	200 (*η*_10_) N/A (*η*_100_)	100	[81]
Ru_0.5_Ir_0.5_O_2_	0.5 M H_2_SO_4_	151 (*η*_10_) 205 ± 10 (*η*_100_)	618.3	[85]
Ru_0.8_Sb_0.2_O_2_	0.5 M H_2_SO_4_	160 (*η*_10_) 238 ± 7 (*η*_100_)	1100	[83]
(RuIr)O_2_/C	0.1 M HClO_4_	174 ± 1.8 (*η*_10_) 237 ± 1 (*η*_100_)	360 (100 mA cm^−2^)	[86]
GB-RuO_2_	0.1 M HClO_4_	187 (*η*_10_) N/A (*η*_100_)	550	[96]
GB-V-RuO_2_	0.5 M H_2_SO_4_	159 (*η*_10_) 222 (*η*_100_)	760	[97]
Zn-RuO_2_	0.5 M H_2_SO_4_	173 (*η*_10_) 227 ± 0.9 (*η*_100_)	1000	[89]
iGa_0.2_Ru_0.8_O_2_	1.0 M HClO_4_	188 (*η*_10_) 219 (*η*_100_)	800 (100 mA cm^−2^)	[92]
Cr_0.6_Ru_0.4_O_2_	0.5 M H_2_SO_4_	220 (*η*_10_) N/A (*η*_100_)	5200 (50 mA cm^−2^)	[93]
RuCo/RuCoO_x_	0.5 M H_2_SO_4_	170 (*η*_10_) 220 (*η*_100_)	2500	[101]
Ru/RuO₂	0.5 M H_2_SO_4_	183 (*η*_10_) 238 ± 10 (*η*_100_)	600	[102]
Er-RuO_x_	0.5 M H_2_SO_4_	200 ± 8 (*η*_10_) 254 ± 10 (*η*_100_)	200	[34]
γ-MnO_2_	1.0 M H_2_SO_4_	489 ± 5 (*η*_10_) 599 ± 15 (*η*_100_)	8000	[105]
Co_2_MnO_4_ on FTO	1.0 M H_2_SO_4_	395 (*η*_10_) 470 ± 10 (*η*_100_)	320 (100 mA cm^−2^)	[107]
Ta_1_Co_3−x_O_4_	0.05 M H_2_SO_4_	378 (*η*_10_) 513 ± 5 (*η*_100_)	140	[109]
CWO-del	0.5 M H_2_SO_4_	288 (*η*_10_) N/A (*η*_100_)	175	[110]
Ni_0.5_Mn_0.5_Sb_1.7_O_y_ film	1.0 M H_2_SO_4_	672 ± 9 (*η*_10_) N/A (*η*_100_)	168	[144]
N-Co_3_O_4_/NC	0.5 M H_2_SO_4_	225 (*η*_10_) 415 ± 10 (*η*_100_)	80	[145]
LaMn-Co_3_O_4_	0.1 M HClO_4_	353 ± 30 (*η*_10_) 453 ± 30 (*η*_100_)	360	[146]
Mn_3_O_4_ nanoplates	0.5 M H_2_SO_4_	210 (*η*_10_) N/A (*η*_100_)	20	[147]
Mn_7.5_O_10_Br_3_	0.5 M H_2_SO_4_	295 ± 5 (*η*_10_) 395 ± 6 (*η*_100_)	500	[148]
C@CeO_2_/Co_3_O_4_	0.5 M H_2_SO_4_	425 (*η*_10_) N/A (*η*_100_)	50	[149]

### Integrated molecular catalysts of the AOER

**Figure 9.**
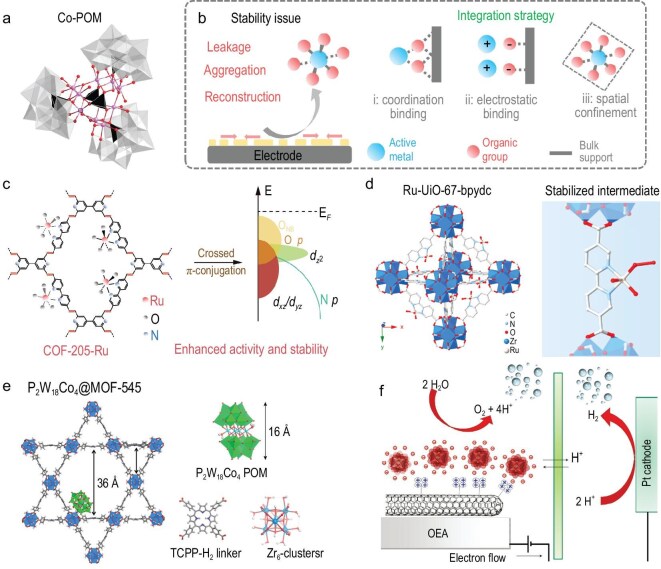
(a) Molecular structure of the [Co_9_(H_2_O)_6_(OH)_3_(HPO_4_)_2_(PW_9_O_34_)_3_]_16_ cluster. Co, pink; O, red; WO_6_, grey octahedra; PO_4_, black tetrahedra. Adapted with permission from Blasco-Ahicart *et al.* [114]; Copyright 2017, Springer Nature. (b) The deactivation and stability challenges and integration strategy of molecular catalysts. (c) COF-205-Ru structure and schematic molecular orbital energy diagram. Adapted with permission from Jia *et al.* [115]; Copyright 2024, Springer Nature. (d) The structure of Ru-UiO-67-bpydc catalyst and corresponding Ru intermediate during the acidic OER by the LOM pathway. Adapted with permission from Yao *et al.* [116]; Copyright 2023, Elsevier. (e) The structure of POM encapsulated in MOF (POM@MOF-545 components). TCPP-H_2_ linker: tetrakis(4-carboxyphenyl) porphyrin. Adapted with permission from Paille *et al.* [117]; Copyright 2018, American Chemical Society. (f) General scheme for a water-splitting electrocatalytic cell with the integrated POM (red)-embedded multi-walled carbon nanotubes surface. Adapted with permission from Toma *et al.* [118]; Copyright 2010, Springer Nature.

Unlike heterogeneous electrocatalysts, molecular catalysts for the OER offer atomistic-level insights into the interaction mechanisms between water molecules and active sites, even though their practical implementation still faces significant challenges. Inspired by the oxygen-evolving complex in natural photosystem II, such as the Mn_4_Ca cubane cluster [111], researchers have developed a diverse series of molecular catalysts for the OER. These catalysts, including mono-, bi- and polynuclear metal complexes, are typically composed of redox-active metal centers (e.g. Fe, Co, Ni, Cu, Ru, Ir etc.) coordinated with organic ligands. Among them, polyoxometalates (POMs) containing active metals such as Co, Ni and Ru have attracted increasing attention as promising homogeneous, Earth-abundant water oxidation catalysts under near-neutral pH conditions [112,113]. The developed Ba[Co-POM]/CP electrodes achieve an overpotential of 189 mV at 10 mA cm^−^² with a catalyst loading of 40% (catalyst/CP) in 0.5 M H_2_SO_4_. Moreover, they maintain relatively stable performance at an overpotential of 250 mV for 1 day (Fig. 9a) [114]. Molecular catalysts serve as model systems to provide atomically well-defined active sites that enable fundamental insights into proton-coupled electron transfer and the formation of O–O bonds. Despite these advantages, molecular catalysts still face significant stability issues under harsh electrochemical conditions, such as leakage, aggregation and reconstruction, which remain critical challenges restricting their direct practical applications (Fig. 9b).

To advance the practical application of molecular catalysts in devices, these integration strategies (such as immobilization, encapsulation and hybridization) have been developed to bridge the gap between molecular catalysts and HEs. Recently, covalent organic frameworks (COFs) have been employed as hosts to immobilize catalytically active metal centers resembling molecular catalysts (Fig. 9c). Atomically dispersed Ru species are anchored in an acid-stable vinyl-linked 2D COF via bipyridine coordination. The crossed π-conjugation tunes the Ru electronic structure, raising the O 2*p* band energy to activate coordinated oxygen, while strong Ru 4*d–*N 2*p* interactions form robust Ru–N bonds, enhancing catalytic stability [115]. In addition to COFs, metal–organic frameworks (MOFs) have also been employed as hosts to immobilize active metal atoms through the functional groups of organic linkers. Atomically isolated Ru oxides are anchored on UiO-67-bpydc via strong coordinating pyridine ligands. The Ru–N bonds formed between Ru oxides and UiO-67-bpydc not only facilitate the participation of lattice oxygen during the OER process but also stabilize the soluble Vo–RuO₄^2^^−^ intermediate (Fig. 9d) [116]. Encapsulation strategies can physically confine active molecular catalysts within porous materials, preventing their leakage and aggregation. By encapsulating Co-POMs within an MOF (MOF-545), the resulting composite acts as a heterogeneous catalyst distinct from molecular catalysts, which exhibits both high activity and good stability for visible-light-driven water oxidation (Fig. 9e) [117]. By tailoring the surface charge of conductive supports, charged homogeneous catalysts can be immobilized. For example, negatively charged POMs can be electrostatically bonded onto the surface of functionalized multi-walled carbon nanotubes. This immobilization strategy enables the construction of relatively efficient and stable nanostructures, thereby bridging homogeneous and heterogeneous catalysis (Fig. 9f) [118]. Researchers have never ceased to draw inspiration from natural photosynthetic systems, and molecular catalysts have provided valuable insights into the evolution of reaction intermediates and the roles of active metal sites during water oxidation. However, even with strategies such as immobilization, encapsulation and hybridization that enable these molecular catalysts to be transformed into relatively efficient and stable heterogeneous structures, their performance under practical electrolyzer conditions remains challenging. Future research will need to further enhance their long-term durability under strongly acidic environments or high current densities, while exploring how to retain the molecular-level tunability and simultaneously achieve efficient integration with heterogeneous systems.

### Artificial intelligence (AI)-accelerated development of HEs for the AOER

The traditional trial-and-error approach has accumulated valuable experience for the design of HEs for the AOER. Data-driven catalyst design powered by AI technologies is emerging as a focal point of research [119–125], offering significant advantages in component optimization, structural screening and the prediction of structure–activity relationships [126,127]. By integrating machine learning algorithms with high-throughput computational and automated robotic platforms, researchers can efficiently navigate the vast design space of catalytic materials. Moreover, AI-assisted models can identify hidden patterns and correlations within complex datasets, enabling the rational design of catalysts with tailored properties [128–131]. As a result, the synergy between AI and traditional methods is expected to drive the next generation of breakthroughs in AOER catalyst development.

**Figure 10.**
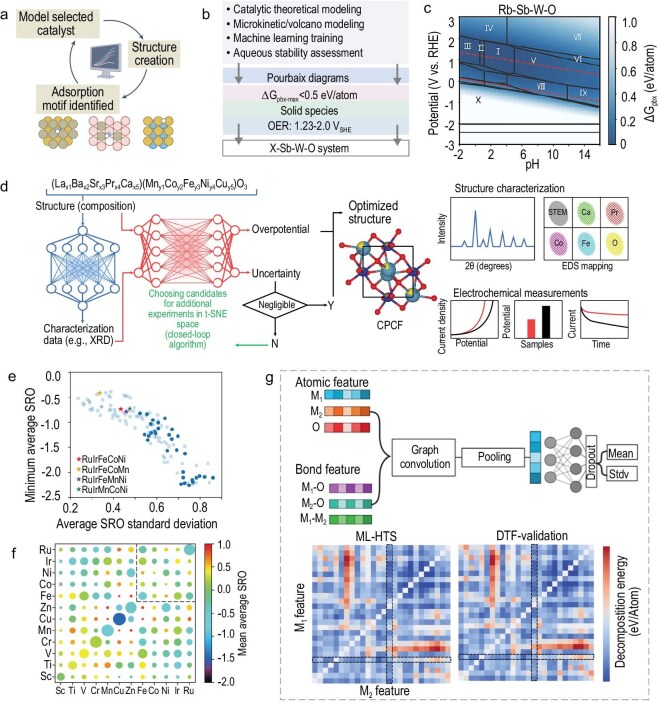
(a) AI-driven design workflow. Adapted with permission from Suvarna *et al.* [134]; Copyright 2024, Springer Nature. (b) Workflow for identifying stable metal oxides in the DigCat platform. (c) Bulk Pourbaix diagram of Rb–Sb–W–O system. Adapted with permission from Jia *et al.* [135]; Copyright 2025, American Chemical Society. (d) The red (prediction) model and the blue (generation) model are individually trained for the screening process, and the predicted optimal oxide is synthesized and undergoes structure characterization and electrochemical performance verification. Adapted with permission from Moon *et al.* [136]; Copyright 2023, Springer Nature. (e) Scatter plot of the minimum average short-range order (SRO) vs. the standard deviation of the average SRO. (f) Correlation plot of the mean (indicated by circle color) and standard deviation (indicated by circle radius) of the average SRO. Adapted with permission from Maulana *et al.* [137]; Copyright 2025, American Chemical Society. (g) Crystal graph convolutional neural network (CGCNN)-HD model heatmaps of Δ*G*_pbx_ and DFT validation stability heatmap for the subset. Adapted with permission from Abed *et al.* [138]; Copyright 2024, American Chemical Society.

Considerable efforts have been devoted to AI-accelerated development of HEs for the AOER, yielding many inspiring and promising results [132,133]. A typical AI-driven design workflow begins with data collection and preprocessing, followed by the extraction of meaningful descriptors that capture the structural and electronic features of candidate materials. Machine learning models are then trained to predict key catalytic properties, such as overpotential or stability, based on these descriptors. Once validated, these models enable high-throughput screening of large material libraries, significantly reducing the time and cost of traditional trial-and-error methods (Fig. 10a) [134]. For structural screening, Jia and co-workers presented a closed-loop framework that predicted RbSbWO_6_ as a promising bifunctional catalyst. Bulk Pourbaix diagrams further indicated its exceptional stability under acidic conditions within the applied OER potential range (Fig. 10b and c). The integrated experimental platform validated the prediction and demonstrated the stable operation of RbSbWO_6_-based heterostructure electrocatalysts in a PEMWE system for 120 h at a current density of 500 mA cm^−2^ [135]. For compositional optimization, AI-driven design offers the advantage of high-throughput screening. The composition of multi-metal oxides, including perovskite oxides, can be effectively optimized through active learning with small datasets, informative structural characterization data and integrated into closed-loop experimental workflows (Fig. 10d). This approach has the potential to yield materials with outstanding catalytic performance [136].

The composition of Ru-based multicomponent alloys was optimized by a machine-learned interatomic potential coupled with replica-exchange molecular dynamics (Fig. 10e and f). The optimized Ru_0.20_(Ir, Fe, Co, Ni)_0.80_ catalyst exhibited improved OER activity and enhanced stability [137]. These acceleration strategies further enhance the design of advanced Ir-based, Ru-based and non-precious-metal-based HEs. For instance, doping strategies have been improved using a machine learning-aided computational pipeline trained on over 36 000 mixed-metal oxides. By rapidly predicting the Pourbaix decomposition energy (G_pbx_), this approach screened 2070 new metallic oxides for their potential stability under acidic conditions. Among them, Ru_0.6_Cr_0.2_Ti_0.2_O_2_ was identified, and its OER activity and stability were experimentally validated (Fig. 10g) [138].

Looking ahead, the continued integration of AI with experimental and theoretical catalysis holds great promise for revolutionizing the design of AOER catalysts. As more high-quality datasets become available and machine learning models grow increasingly sophisticated, we can expect more accurate predictions and deeper insights into catalytic behavior at the atomic scale. Ultimately, this interdisciplinary approach will not only expedite the discovery of efficient and stable catalysts but also contribute to the broader goal of achieving sustainable and scalable hydrogen production through advanced water electrolysis technologies.

## CONCLUSIONS AND PERSPECTIVES

The AOER plays a vital role in various electrochemical and energy-related technologies, particularly in PEMWEs. This review profiles significant research efforts and representative design strategies for both Ir- and Ru-based and non-noble-metal (Mn, Co)-based HEs towards the AOER. Reviewing the underlying mechanisms, structure–activity relationships and advanced material design strategies helps us overcome the intrinsic challenges of the AOER, such as sluggish reaction kinetics, limited catalyst stability and the high cost associated with noble metals. Moving forward, future catalyst design should meet the industrial requirements of PEMWEs. Some key issues should be considered and are summarized as follows. Tackling these challenges will help accelerate the widespread implementation of PEMWEs and other electrochemical energy technologies, including the following four aspects.

- *Minimizing usage without compromising performance for Ir-based HEs*. Ir-based HEs remain the primary choice for the industrial application of PEMWEs. Their intrinsic ultrahigh stability and excellent activity ensure reliable large-scale hydrogen production. However, the widespread deployment of PEMWEs demands stringent cost control, which in turn calls for maximizing the utilization of Ir. Reducing Ir loading must not come at the expense of catalytic activity and stability. This imposes stringent requirements on catalyst design, catalyst ink formulation and MEA fabrication processes, necessitating a deeper understanding of the HEs from the microscopic to macroscopic levels.
- *Enhancing stability and elucidating the mechanism for Ru-based HEs*. Promising Ru-based HEs are being actively developed. Enhancing their stability remains the primary challenge. Equally important is understanding the origins of improved stability and elucidating the underlying mechanisms, which are crucial for uncovering the true nature of solid-state HEs and guiding the development of more robust HEs. Achieving this requires deeper conceptual insight and the application of advanced *in situ* characterization techniques. More importantly, Ru-based HEs must be further pushed toward integration into PEMWE devices. Investigating the *in situ* evolution of Ru-based MEAs under realistic operating conditions will be especially meaningful for evaluating their practical viability.
- *Expanding the potential stability window of non-noble-metal HEs.* γ-MnO₂ operates stably at low potential, and the resulting current densities fall short of the industrial requirements for PEMWEs. However, recent advances are encouraging. By enhancing structural stability and reducing the AOER overpotential, stable operation at higher current densities is becoming achievable. It is highly anticipated that non-noble-metal-based HEs will be able to operate stably at high current densities in PEMWEs.
- *Developing advanced characterization techniques.* The OER involves complex multi-electron transfer, multiple intermediate species, and surface reconstruction of the catalyst, which place higher demands on *in situ* characterization techniques to capture dynamic structural, electronic and surface changes under realistic electrochemical conditions. Emerging approaches, including high-resolution spectroscopy, synchrotron-based techniques and spatially resolved imaging, provide unprecedented insights into local atomic configurations, metal–oxygen interactions and transient intermediates. Moreover, integrating these experimental techniques with data-driven and AI-assisted analyses can accelerate the elucidation of structure–activity–stability relationships. Investigating OER mechanisms across different types of catalysts further enables rational catalyst design to overcome the inherent trade-off between activity and stability.
- *AI and automated robotic platforms for OER.* Looking ahead, the profound integration of AI and automation technologies will chart an unprecedented course for the research of HEs of OER. AI, leveraging its formidable data processing and model construction capabilities, can meticulously sift through vast material databases to pinpoint catalyst structures with latent superior performance, drastically slashing the protracted trial-and-error cycle of traditional methods. Meanwhile, automated robotic platforms enable seamless end-to-end integration of experimental design, sample preparation and performance testing, ensuring both the efficiency and accuracy of data collection.

**Figure 11.**
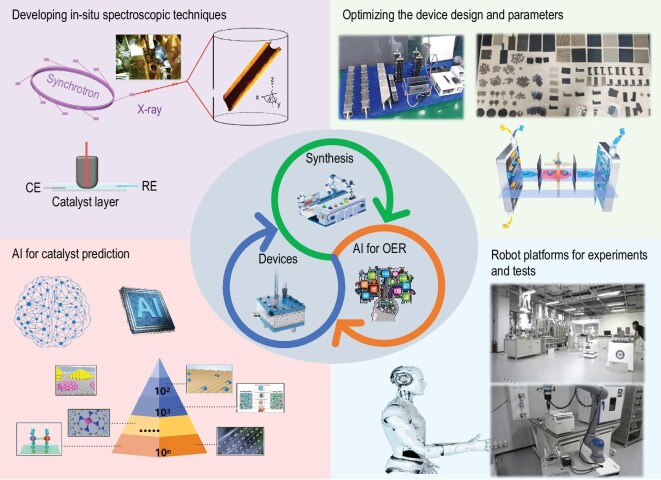
Future roadmap for the design and optimization of HEs of the AOER including: *in situ* spectroscopic techniques to enhance fundamental understanding; optimization of industrial parameters to enable large-scale application of PEMWEs-based hydrogen production; and AI-assisted catalyst prediction and robotic platforms for high-throughput experiments to accelerate the development of active, durable and cost-effective HEs.

To address these challenges, more advanced technologies involving chemistry, mechanical engineering and computer science deserve our attention. From a deeper understanding of OER at the microscopic level, to the development of nanocatalysts, and further to the optimization of MEA process parameters, the advanced industrial PEMWE hydrogen production relies heavily on progress in emerging technologies. In the future roadmap, it is foreseeable that advanced technologies such as AI for catalyst prediction and robotic platforms for automated experiments and testing will significantly accelerate the development of highly active, durable and cost-effective HEs of the AOER. Moreover, the development of *in situ* spectroscopic techniques, along with the optimization of device design and operational parameters, will contribute to fundamental understanding at the microscopic scale and large-scale industrial applications of PEMWE-based hydrogen production (Fig. 11).
